# Higher Pregnancy Success Rates in Patients with Diminished Ovarian Reserve < 40 Years When Initially Treated by Intrauterine Insemination with Mild Ovarian Stimulation Compared to In Vitro Fertilization Alone: A Pilot Study

**DOI:** 10.1007/s43032-025-01830-w

**Published:** 2025-03-25

**Authors:** Julia J. M. Deneer, Saskia Le Cessie, Evert J. P. van Santbrink, Lucette A. J. van der Westerlaken, Eileen E. L. O. Lashley

**Affiliations:** 1 Department of Obstetrics and Gynecology, Leiden University Medical Center, Post Zone H-03, Route 485, Albinusdreef 2, Leiden, ZA 2333 the Netherlands; 2 Department of Clinical Epidemiology, Leiden University Medical Center, Leiden, the Netherlands; 3 Department of Reproductive Medicine, Reinier de Graaf Hospital, Voorburg, the Netherlands

**Keywords:** Diminished ovarian reserve, Artificial reproductive technology, Pregnancy rates, Pregnancy outcomes, Instrumental variable analysis

## Abstract

For women with idiopathic diminished ovarian reserve (DOR), direct start with IVF has been suggested to potentially shorten the time to pregnancy. Others however prefer intra-uterine insemination with ovarian stimulation (IUI + OS) due to the expected low response in IVF. In this pilot study, we determined the effect of these two strategies in women with DOR < 40 years. From a retrospective cohort, we included 135 women that met the diagnostic criteria of DOR. Patients were randomly referred to two different outpatient clinics in the Netherlands between 2012–2018 because of subfertility. Primary outcome was clinical pregnancy; secondary outcomes included ongoing pregnancies, live births, time to pregnancy and pregnancy-related complications. An instrumental variable analysis was used to assess the average effect of treatment with IUI + OS followed by IVF (protocol A) compared to IVF alone (protocol B) and correct for (unknown) confounders. Treatment protocol A was performed in 72.6% patients in Centre 1 and 30.6% in Centre 2. In Centre 1 61.6% (45/73) women had a clinical pregnancy compared to 41.9% (26/62) in Centre 2 (difference 19.7% (95% CI 3.1%-36.3%), *p* = 0.02). Early miscarriage occurred in 24% of the women in Centre 1 in comparison to 45% of the women in Centre 2. There were no significant differences in pregnancy-related complications. This pilot study suggests that women < 40 with DOR, if treated with IUI + OS followed by IVF, have higher estimated cumulative clinical pregnancy success, with a trend towards higher ongoing pregnancies and live births, in comparison to women treated with IVF alone.

## Introduction

### Background

A relevant contribution for female subfertility is represented by a diminished ovarian reserve (DOR) [1]. With the decrease of the ovarian reserve, encompassing both the quantity and quality of primordial follicles, the ovary loses its normal reproductive potential [2]. In literature different terminology exists to refer to this diminished ovarian pool [3, 4] and various criteria and cut-off levels of ovarian reserve tests are used for the definition [5, 6]. Based upon these varying terms and cut-offs, a reliable incidence of DOR in women who wish to conceive is currently unknown. The incidence of poor ovarian response, a surrogate measure of DOR, ranges from 9 to 24% for all assisted conception cycles [7].

Currently, there is no known fertility strategy that has reliably showed an increase in ovarian activity and natural conception rate in women with low ovarian reserve [8–11]. To decrease further reduction of the ovarian pool, direct start with in vitro fertilization (IVF) has been suggested to potentially shorten the time to pregnancy. Women with low ovarian reserve however often respond poorly to controlled ovarian stimulation resulting in retrieval of fewer oocytes, producing poorer quality embryos and reduced implantation- and pregnancy rates [12]. Intra-uterine insemination in combination with ovarian stimulation (IUI + OS) is considered an effective treatment for unexplained fertility or mild male subfertility. The risk for complications is lower and the treatment is less invasive compared to IVF [13, 14]. The question is therefore which method, or strategy of artificial reproductive technology will result in a higher pregnancy rate in women with DOR and secondary, has the lowest risk for (pregnancy) complications and lowest costs. To compare these two different treatment strategies, in general the randomized controlled trial is considered the gold standard study design. However, in the face of financial and practical challenges (for example the low estimated incidence of DOR and thus a small study population), the observational study design is more realistic. In this pilot study we focus on clinical outcomes by using the instrumental variable analysis to achieve pseudo-randomisation, enabling comparison of the different treatment policies [15, 16].

## Materials and Methods

### Study Design, Setting and Participants

We conducted a retrospective cohort study, including women under 40 years referred to the outpatient clinic of two hospitals, between January 2012 and December 2018 with idiopathic DOR. The definition of DOR used in this study, based on literature and expert opinion, is characterized by (a) subfertility and (b) an elevated early follicular FSH level (≥ 10 IU/L), in combination with low estradiol serum concentration (≤ 150 pmol/L), and/or low AMH (below fifth percentile on an age-depended scale) [17] and/or low antral follicle count (AFC)(< 7) [5, 18, 19]. Exclusion criteria for this study were age > 40 years at time of diagnosis DOR, iatrogenic cause of DOR, women with irregular cycle (cycle length above 35 days or below 21 days), women that applied to oocyte donation and couples with one or more of the following criteria (a) two blocked or absent fallopian tubes, (b) absolute contraindication for twin gestation or (c) total motility count of sperm < 3 million.

Both hospitals operate as independent IVF centers with comparable numbers of treatment and ovum pick-up. The centers are closely situated in the southern part of the Netherlands. The Netherlands has an unique health care system where referral of patients to a center is randomly allocated by the general practitioner based on patients zip code and not based on patients preferences. Therefore, we assume a similar distribution of prognostic factors related to the outcomes.

The study was approved by the Committee of Medical Ethics of both centers (reference number G19.059).

### Variables and Data Sources

In this study, we analyzed whether the pregnancy success rates and incidence rates of (pregnancy) complications differed between treatment protocol A; intra uterine insemination (IUI) in combination with ovarian stimulation, followed by IVF (if not successful after maximum of 6 cycles IUI), or treatment protocol B, IVF alone (see Appendix 1). Centers are allowed to perform their preferred treatment protocol as there is no (inter)national consensus of the best treatment strategy.

We performed an ‘intention to treat’ analysis; women who started with protocol B, but had an escape IUI, were analyzed in the treatment B group.

Patients were identified through electronic files of all patients who received fertility treatment between January 2012 and December 2019 in Centre 1 and 2. All data variables were collected from these files and documented in a research Castor database.

### Outcomes

The primary outcome of this study was clinical pregnancy within 24 months after start of fertility treatment, defined as the presence of a gestational sac on transvaginal ultrasound at 6–8 weeks of gestational age. Secondary outcome measures of this study are pregnancy success rates and time until first clinical pregnancy. Pregnancy success includes ongoing pregnancy rate, live birth rate and time to clinical pregnancy. Ongoing pregnancy is defined as vital pregnancy beyond 12 weeks' gestation. Live birth is defined as the delivery of one or more living infant(s) after 24 weeks' gestation. We considered these outcomes binary, meaning that ≥ 1 pregnancy within the same patient was counted as 1. Spontaneous pregnancies were involved in the analysis to provide an intention to treat analysis.

In addition, complications were considered as secondary outcome measures, including treatment complications (ovarian hyperstimulation, cycle cancellation, poor response (2 or less dominant follicles > 12 mm growing with IVF treatment), complications due to ovum pick-up) and pregnancy complications (miscarriages, ectopic pregnancy, multiple pregnancy, hypertensive complication, growth restriction and preterm labor).

### Statistical Analyses

The study was performed as a pilot study, enabling sample size calculation in a subsequent larger and more comprehensive future trial. While there is no definitive rule or formula for calculating the optimal sample size for a pilot study, we followed the recommendation by Whitehead et al. to use a sample size of 75 to 25 patients, when the standardized effect sizes are extra small (0.1) or small (0.2) [20].

Statistical analyses are performed using SPSS Statistics 25 (IBM SPSS Software) and R version 4.2.0 with packages sem and boot, to perform the IV-analysis. To analyze differences in clinical characteristics between the two centers, unpaired t-tests is used for continuous data and the Chi-square test for categorical data. Time until first clinical pregnancy is compared between the two centers using Kaplan Meier curves and the Log Rank test. For all tests a two-sided p-value < 0.05 or 95% confidence interval not including the null value, indicates a statistically significant outcome.

Since treatment protocol A is mainly performed in Centre 1 and treatment protocol B is the strategy of choice in Centre 2, we performed an “instrumental variable analysis” (IV-analysis). With this analysis the treatment of the patient is considered ‘randomly’ allocated by simply visiting the treating center that the patient is referred to. An instrumental variable is a variable which is related to exposure, only related to the outcome through exposure and independent of the unmeasured confounders. The IV-analysis is a technique that removes the effect of hidden bias and, in comparison to the propensity score, does not assume that all potential confounders are observed. The results of the IV-analysis show the difference in outcome and should in this study be interpreted as the estimated effect of the treatment protocol on the investigated outcome. In our study, the center of treatment is the IV. The IV-analysis is performed using a twostep linear regression, with 95% confidence intervals calculated by percentile bootstrapping [15, 16].

## Results

### Baseline Characteristics

A total of 135 patients referred because of subfertility and diagnosed with DOR between 2012 and 2019, were included in this study; 73 patients were included from Centre 1 and 62 patients from Centre 2. There was no loss to follow-up (see for flowchart Figure 2 in Appendix 2 Fig. 2). Table 1 shows the patient characteristics and hormonal profiles at baseline of the two groups. We did not observe any differences between women treated in Centre 1 in comparison to Centre 2, except for social economic status. The hormonal profiles at baseline did not differ between the centers, however several measurements of AMH and AFC were missing.

**Table 1:** Patient characteristics and hormonal profiles at baseline

	Centre 1 (*n* = 73)	Centre 2 (*n* = 62)	*p*-value
Age at diagnosis (y)	34.7 ± 3.3	35.5 ± 3.1	ns
Duration of subfertility before diagnosis (m)	28.3 ± 17.9 (*n* = 72)	24.1 ± 21.0 (*n* = 62)	ns
BMI (kg/m^2^)	24.0 ± 3.5	24.0 ± 3.6	ns
Smoking (*n*)	9 (12.3%)	11 (17.7%)	ns
Socioeconomic status (SES)	2.14 ± 0.8	2.40 ± 0.6	0.05
Primary infertility (*n*)	33 (45%)	36 (58%)	ns
Day 3 Follicle Stimulating Hormone (FSH) (IU/L)	14.9 (IQR 12 – 17) (*n* = 72)	15.1 (IQR 11.5 – 18.5) (*n* = 57)	ns
Day 3 Estradiol (pmol/L)	101.4 (IQR 70 −130) (*n* = 72)	106.7 (IQR 55 −132) (*n* = 55)	ns
Anti-Mullerian Hormone (AMH) (ng/mL)	0.6 (IQR 0.2 – 0.8) (*n* = 72)	0.1 (IQR 0.1 – 0.8) (*n* = 17)	ns
Antral Follicle Count (AFC) (mean) (*n*)	5 (IQR 3 – 7) (*n* = 62)	4 (IQR 3 – 6) (*n* = 55)	ns
Menstrual cycle pattern (*n*)			
< 21 days 21–35 days > 35 days or amenorrhea	2 (2.7%) 65 (89%) 6 (8.3%)	4 (6.4%) 51 (82.3%) 7 (11.3%)	ns

The scale is ranging from −1 to 3, with −1 being the lowest and 3 the highest score

y = years

m = months

n = total amount of women (to indicate the amount of missing data)

±  = standard deviation

IQR = interquartile range

SES = Based on average education and income per zip code in Netherlands in 2017

ns = not significant

### Treatment Protocol

The majority of women in Centre 1 was treated according to treatment protocol A; 53 women (72.6%). As expected, these rates were significantly different (*p*-value < 0.001) from Centre 2, where 19 women (30.6%) were treated according to treatment protocol A **(**Table 2). Regarding protocol adjustments; a total of 25 women that were planned for IVF (in protocol A or B) switched to an escape IUI. This was similarly divided between the two centers and none of the women made a permanent switch of treatment protocol.

**Table 2:** Distribution of treatment

	Centre 1 (*n* = 73)	Centre 2 (*n* = 62)	*p*-value
Treatment protocol A (*n*) Treatment protocol B (*n*)	53 (72.6%) 20 (27.4%)	19 (30.6%) 43 (69.4%)	*p* < 0.001
Treatment adjustments			
Escape IUI after IVF (*n*)	13	12	
Cancellations of IUI (*n*)			
Low response	0	1	
Hyperstimulation	3	3	
Cancellations of IVF(*n*)	9	8	
Total fertilization failure (*n*)	12	2	
OHSS (*n*)	0	0	
Total amount of treatments per person Total amount overall	4.0 (median) 305	2.5 (median) 196	ns
Ovarian stimulation			
Ovarian stimulation with IUI Controlled ovarian hyperstimulation with IVF GnRH treatment	anti-estrogen 50 mg or rec-FSH 50/75 IE rec-FSH 150–225 IE GnRH-antagonists	anti-estrogen 50 mg or rec-FSH 50/75 IE rec-FSH 150–225 IE GnRH-agonists	

(*n*) = Total amount of women

ns = not significant

The ovarian stimulation in protocol A was in both centers performed with the use of anti-estrogen, mostly clomiphene citrate, or recombinant FSH (rec-FSH). The treatment in protocol B differed; in Centre 1 mainly gonadotropin releasing hormone (GnRH) antagonists are used in contrast to the use of mainly GnRH-agonists in Centre 2.

### Primary Outcome

Clinical pregnancy within 24 months occurred in 45/73 (61.6%) women in Centre 1, compared to 26/62 (41.9%) in Centre 2 (difference 19.7% (95% CI 3.1%−36.3%), *p* = 0.02). The IV estimate of the difference in percentage was 46.9% (10.2%−70.6%) [21]. This should be interpreted as follow: the estimated effect is that 46.9% more women have a clinical pregnancy when treated according to protocol A in comparison to protocol B. The results are shown in Table 3.

**Table 3:** Reproductive outcomes

	Centre 1 (*n* = 73)	Centre 2 (*n* = 62)	Mean difference (95% CI)	Estimated mean difference IV-analysis	*P*-value (Chi-square test)
Women with ≥ 1 clinical pregnancies Total pregnancies	45 (61.6%) 53	26 (41.9%) 40	19.7% (3.1%−36.3%)	46.9% (10.2%−70.6%)	0.02
Women with ≥ 1 ongoing pregnancies Total pregnancies	37 (50.7%) 41	25 (40.3%) 31	10.4% (−6.4%, 27.1%)	24.7% (−20.5%, 53.2%)	0.23
Live births Total live births	37 (50.7%) 44*	22 (35.5%) 28 **	15.2% (−1.3%, 31.7%)	36.2% (−5%, 62.6%)	0.08

A total of 16 women, four from Centre 1 and 12 from Centre 2, conceived spontaneously. In both centres, for 4 women this spontaneous clinical pregnancy was the only pregnancy during the observed 24 months, whilst the other 8 women conceived spontaneously after their first clinical pregnancy conceived after fertility treatment

^*^ One twin and one triplet pregnancy, resulting in 44 live births out of 41 ongoing pregnancies

^**^ One twin pregnancy and four late miscarriages (termination of pregnancies), resulting in 28 live births out of 31 ongoing pregnancies

### Secondary Outcomes

Next to the clinical pregnancy rate, we calculated the estimated average effect of treatment with protocol A for ongoing pregnancy and live birth rate 24 months after start of treatment. In Centre 1, 37/73 (50.7%) women had at least one ongoing pregnancy in comparison to 25/62 (40.3%) women in Centre 2 (difference 10.4% (−6.4%, 27.1%)). This results in an IV estimated effect of 24.7% (10.4/0.42). These pregnancies led to a total amount of 37/73 (50.7%) versus 22/62 (35.5%) women with one or more live births, respectively (difference 15.2% (−1.3%, 31.7%)). This results in an estimated effect of 36.2% (15.2/0.42)**.** All outcomes are shown in Table 3**.**

**Fig. 1:**
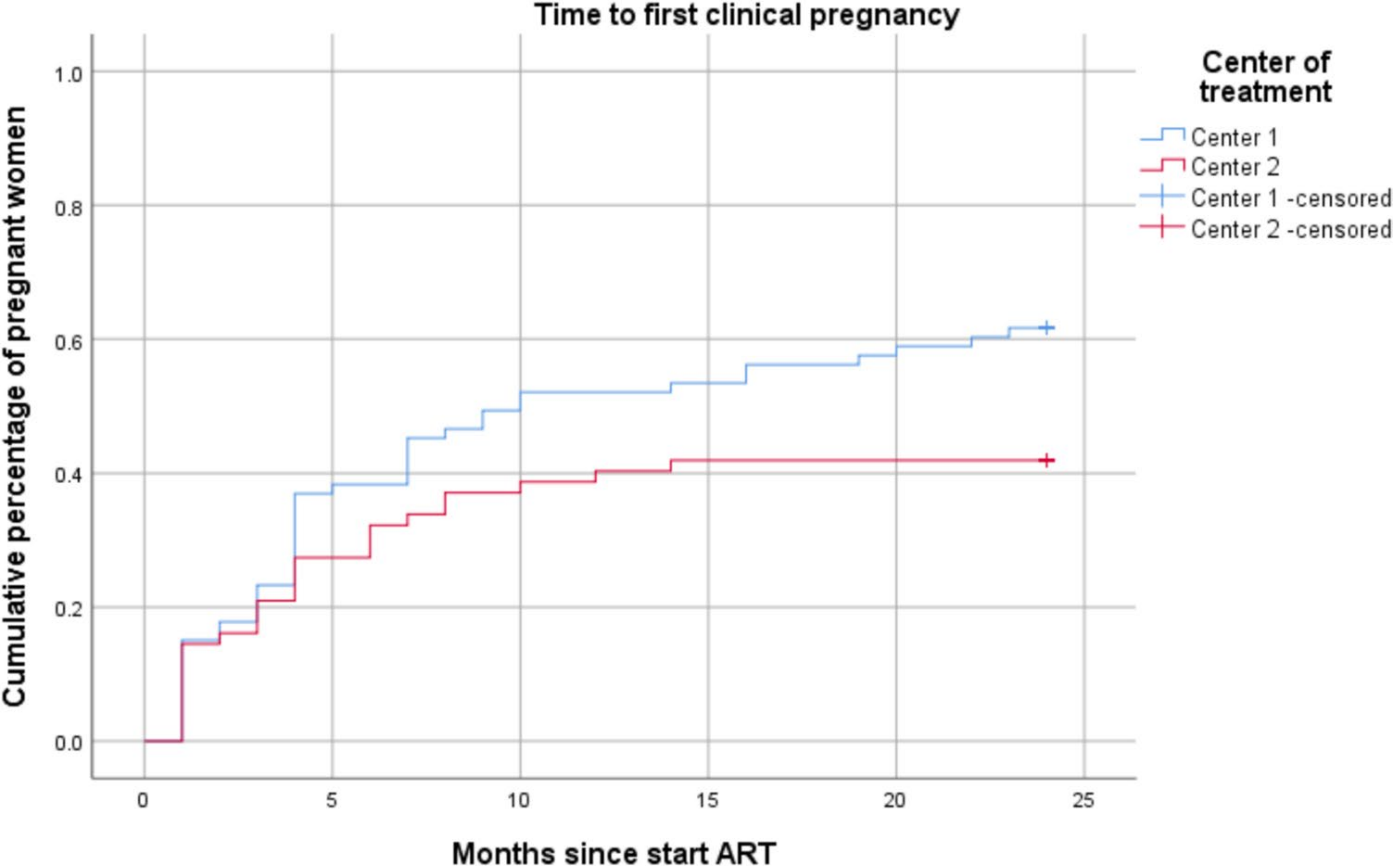
Kaplan Meier curves to compare time to first clinical pregnancy according to center of treatment. Blue line = Centre 1. Red line = Centre 2

**Fig. 2:**
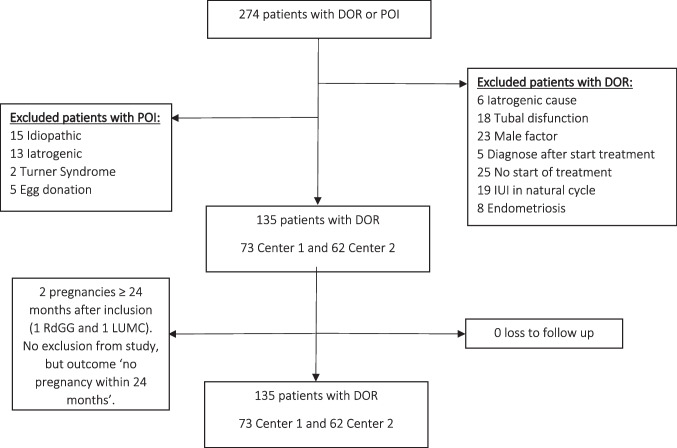
Flowchart of patient inclusion

Kaplan Meier curves to compare time to the first clinical pregnancy between the two different centres are shown in Fig. 1. After 10 months 50% of the women in Centre 1 achieved their first clinical pregnancy, while in Centre 2 only 41.9% of the women conceived during the 24 months of follow-up. The differences were statistically significant (Log Rank *p* = 0.046).

### Pregnancy Complications

In Centre 1, 11/46 (23.9%) of women with a biochemical pregnancy had an early miscarriage in comparison to 14/31 (45.2%) women in Centre 2 (difference 21.3% (95% CI −5.8% – 20.8%)). All other pregnancy-related complications and neonatal outcomes are summarized in Table 4.

**Table 4:** Pregnancy-related complications and neonatal outcomes

	Centre 1 (*n *= 46)	Centre 2 (*n *= 31)	Estimated mean difference IV-analysis	*P*-value (Chi-squared)
Early miscarriage (*n*)	11(23.9%)	14 (45.2%)	−50.7%	0.27
Late miscarriage (*n*)	0	4*	-	-
Ectopic pregnancy (*n*)	2**	3***	-	-
Hypertensive complications (*n*)	3	1	-	-
Twin gestation after IVF (*n*)	1	1	-	-
Triplet gestation after IUI (*n*)	1	0	-	-
Neonatal outcomes				
Prematurity Dysmaturity Macrosomia NICU	3 3 1 3	2 1 1 1	-	-

(*n*) = Total amount of women

* = Termination of pregnancy because of a) two cases of trisomy 21 b) severe heart disease c) spontaneous late miscarriage

** = One ectopic pregnancy after artificial reproductive technology and one after spontaneous pregnancy

*** = Two ectopic pregnancies after artificial reproductive technology and one after spontaneous pregnancy

## Discussion

The current study examined reproductive outcomes in women under 40 with idiopathic DOR after treatment with IUI + OS followed by IVF (protocol A) compared with IVF treatment alone (protocol B). We hypothesized that treatment with IUI + OS followed by IVF would result in higher pregnancy rates, due to the higher number of treatment cycles, in combination with the beneficial opportunity of finishing treatment even while low ovarian response occurs. Indeed, the IV analysis showed a higher clinical pregnancy rate for women treated with protocol A compared to protocol B, with an estimated effect of 46.9%. This should interpreted as an estimated effect of 46.9% more women with a clinical pregnancy when treated with protocol A in comparison to protocol B. Regarding the secondary outcomes, we also observed a trend towards higher ongoing pregnancy rate, higher live birth rate and shorter time to pregnancy. Moreover, a higher early miscarriage rate was found in women treated with protocol B.

A number of previous studies reported on reproductive outcomes after IUI + OS or IVF with different stimulation protocols in women with DOR. Ongoing pregnancy rates varied between 16.8% to 32% and live birth rate between 13.4% to 30.3% [22–25]. In most of these studies however, different definitions of DOR are used, not corresponding with the definition used in our study. This could explain the difference in reproductive outcomes as observed in our study. Another explanation is the different duration of the follow-up in mentioned studies. In our study the outcomes were evaluated 24 months after start with ART, whilst the summarized studies evaluated up to 12 months maximum. We decided to continue follow-up until 24 months, since the treatment protocol could include a maximum amount of 6 cycles of IUI + MOH followed by 3 cycles of IVF. This could take at least 12 months, but with expected breaks and cancellations this can take up to 24 months (Fig. 1). Finally, we included spontaneous pregnancies as well, to show a representative percentage of pregnancy outcomes for women with idiopathic DOR.

Studies comparing the effect of different types of fertility treatment (IUI + OS versus IVF) or combinations of ART (IUI + OS followed by IVF) in women with idiopathic DOR are currently lacking. There is however considerable evidence on the treatment of women with unexplained subfertility. Bensdorp et al. randomized couples between IVF with single embryo transfer, IVF in a modified natural cycle or IUI + OS. The clinical pregnancy rates within 12 months varied between 59–67%, and live birth rates between 51–59% [13]. Nandi et al. compared pregnancy rates after either three cycles of IUI + OS or one cycle of IVF within a time frame of 6 months from randomization. They showed a clinical pregnancy rate of 33.6% after IUI + OS and 46.2% after IVF [26]. Interestingly, though we only selected women with DOR and subfertility, the pregnancy rates in our study are comparable to the unexplained subfertile population in these studies. Possibly, some patients with unexplained subfertility could suffer from undiagnosed DOR. On the other hand, whilst the results are comparable, the time to follow-up was only 6 months in comparison to 24 months in our study. The low ovarian reserve and expected low response during treatment in our population might explain this observation and confirms that time to pregnancy is longer in patients with DOR. This again indicates that the number of fertility treatments and thus the time to follow up are a cause of the outcomes in this study in favour of treatment with protocol A.

Obviously, an important limitation of this study is its observational design and hence the risk of bias by confounding-by-indication. We decided to perform an IV-analysis, since this method yields an asymptotically unbiased estimate of the treatment effect in the subgroups of compliers, even in the presence of unmeasured confounding, for example confounding by indication. Obtaining a valid IV-analysis requires however fulfilment of three assumptions [15]; The first assumption is existence of correlation between the IV (centre) and the exposure (treatment). Our study demonstrates that allocation of treatment with IUI + OS followed by IVF is 72.6% in Centre 1 and 30.6% in Centre 2. This is sufficient to perform a valid IV-analysis [21, 27].

The second assumption requires that there is no relationship between the instrument and the (unmeasured) confounders, for example by differences in baseline characteristics of the patient population in the two centres. For measured confounders, we only observed a nearly significantly difference regarding the socioeconomic status (SES). Both centres show a SES above average, which is often seen in patients receiving ART. However, in this study a higher SES seems not to be associated with better outcomes, since patients in Centre 2 have a higher SES but the outcomes are in favour of Centre 1. Although baseline characteristics are comparable, there could exist unknown differences between the populations of the two centres, explaining the results. The third assumption states that there is only a relationship between the IV and the outcome by its relationship with the exposure and not by other variables, neither directly nor indirectly. This means that method of treatment in both centres should be comparable and no major differences in other aspects of care are present. In this study, both anti-estrogen as rec-FSH was used as ovarian stimulation in IUI + OS, with a preference for rec-FSH in Centre 1. Literature shows that in couples with unexplained subfertility an ovarian stimulation regimen with rec-FSH compared to clomiphene citrate results in similar pregnancy rates if adherence to strict cancellation criteria to prevent multiple pregnancies is maintained [28]. The ovarian stimulation during IVF treatment was different in both centres: in general, a GnRH-antagonist was used in Centre 1 and a GnRH-agonist in Centre 2. Whether the outcomes of our IV-analysis are the result of this difference seems unlikely, as previous studies demonstrated non-significant clinical differences in reproductive outcomes between different ovarian stimulation protocols [22, 24]. In summary, violation of these assumptions can be a threat for the validity of the IV-analysis [15]. Despite the fact this concern appears to be unlikely in this study, it is important to consider and deliberate whilst interpreting the results.

A strength of our study is the fact that this a well-defined cohort of women with strict in- and exclusion criteria. With respect to classification of the women into one of the fertility treatment groups, we included all performed fertility treatments up to maximum one year before diagnosis was officially made in order to prevent selection bias. This decision can be explained by the fact DOR is a non-acute disease and diagnosis was occasionally made during fertility treatment, for example after poor response to ovarian stimulation.

In conclusion, the present study suggests that women < 40 years with DOR, if treated with IUI + OS followed by IVF, have higher estimated chances of achieving clinical and ongoing pregnancy, and live birth in comparison to women treated with IVF alone. A larger, multicentre prospective study is recommended to investigate these promising results, taking also miscarriage rates, cost-effectiveness and psychological impact into account. In addition, we recommend the use of a clear, universal definition of DOR for the generalizability of these future results.

## Data Availability

Not applicable.

## Appendix 1

### Description of Different Treatment Protocols

*Treatment protocol A*, mainly performed in Centre 1, consisted of ovarian stimulation (OS) by urinary or recombinant gonadotrophins with starting dose of 50 or 75 IU or anti-estrogen 50 mg or 100 mg daily. Patients were monitored by ultrasound with cancellation criteria at > 3 follicle ≥ 12 mm. In case of this cancellation or with monofollicular growth, the dose was adjusted in the subsequent cycle. Semen samples were processed within one hour of ejaculation according to local protocol and women were inseminated 36 to 40 h after hCG injection. IUI was performed once per cycle, a maximum of 6 cycles were performed. If not successful, in vitro fertilization (IVF) was performed, according to treatment protocol B.

*Treatment protocol B*, mainly performed in Centre 2, consisted of an IVF treatment according to standard regimens with ovarian stimulation by urinary or recombinant gonadotrophins, day 1 agonist protocol or day 6 GnRH antagonist protocol. Final oocyte maturation was achieved by hCG injection if ≥ 3 follicles of at least 17 mm were present. If not, the cycle was cancelled or converted into an escape IUI. An escape IUI is considered part of the treatment and will therefore not be assigned as a switch of treatment. IVF treatment is cancelled definitely when there is repeatedly no response after stimulation. Embryo Transfer was on day 3 and luteal phase supplementation consisted of 600 mg natural micronized progesterone in three separate doses starting one day after oocyte retrieval and continued until 18 days after ovum pickup.
